# Validity and Reliability of Fitbit Flex for Step Count, Moderate to Vigorous Physical Activity and Activity Energy Expenditure

**DOI:** 10.1371/journal.pone.0161224

**Published:** 2016-09-02

**Authors:** Ashleigh Sushames, Andrew Edwards, Fintan Thompson, Robyn McDermott, Klaus Gebel

**Affiliations:** 1 College of Healthcare Sciences, James Cook University, Cairns, Queensland, Australia; 2 Centre for Chronic Disease Prevention, College of Public Health, Medical and Veterinary Sciences, James Cook University, Cairns, Queensland, Australia; 3 Faculty of Sport and Health Sciences, University of St Mark and St John, Plymouth, Devon, England; 4 Prevention Research Collaboration, Sydney School of Public Health, University of Sydney, Sydney, New South Wales, Australia; Vanderbilt University, UNITED STATES

## Abstract

**Objectives:**

To examine the validity and reliability of the Fitbit Flex against direct observation for measuring steps in the laboratory and against the Actigraph for step counts in free-living conditions and for moderate-to-vigorous physical activity (MVPA) and activity energy expenditure (AEE) overall.

**Methods:**

Twenty-five adults (12 females, 13 males) wore a Fitbit Flex and an Actigraph GT3X+ during a laboratory based protocol (including walking, incline walking, running and stepping) and free-living conditions during a single day period to examine measurement of steps, AEE and MVPA. Twenty-four of the participants attended a second session using the same protocol.

**Results:**

Intraclass correlations (ICC) for test-retest reliability of the Fitbit Flex were strong for walking (ICC = 0.57), moderate for stair stepping (ICC = 0.34), and weak for incline walking (ICC = 0.22) and jogging (ICC = 0.26). The Fitbit significantly undercounted walking steps in the laboratory (absolute proportional difference: 21.2%, 95%CI 13.0–29.4%), but it was more accurate, despite slightly over counting, for both jogging (6.4%, 95%CI 3.7–9.0%) and stair stepping (15.5%, 95%CI 10.1–20.9%). The Fitbit had higher coefficients of variation (C_v_) for step counts compared to direct observation and the Actigraph. In free-living conditions, the average MVPA minutes were lower in the Fitbit (35.4 minutes) compared to the Actigraph (54.6 minutes), but AEE was greater from the Fitbit (808.1 calories) versus the Actigraph (538.9 calories). The coefficients of variation were similar for AEE for the Actigraph (C_v_ = 36.0) and Fitbit (C_v_ = 35.0), but lower in the Actigraph (C_v_ = 25.5) for MVPA against the Fitbit (C_v_ = 32.7).

**Conclusion:**

The Fitbit Flex has moderate validity for measuring physical activity relative to direct observation and the Actigraph. Test-rest reliability of the Fitbit was dependant on activity type and had greater variation between sessions compared to the Actigraph. Physical activity surveillance studies using the Fitbit Flex should consider the potential effect of measurement reactivity and undercounting of steps.

## Introduction

The health benefits of physical activity are well established [1]. The current Australian physical activity guidelines recommend for adults to accumulate at least 150 to 300 minutes of moderate intensity activity or 75 to 150 minutes of vigorous activity per week [2]. Based on self-report it has been estimated that up to 60% of Australian adults do not meet the recommended activity levels [3]. However, self-reported measures of physical activity can be inflated due to recall and social desirability bias [4]. For instance, based on accelerometry, a study in the USA found significantly lower compliance rates with activity guidelines than previously estimated through self-reported activity [5].

Physical activity can be assessed using a variety of validated techniques. Methods such as direct observation and doubly labelled water can provide useful information, however, they are associated with an increased participant burden and a high cost [6]. One of the most extensively validated accelerometers utilised to measure physical activity is the Actigraph GT3X+ [7, 8], a triaxial accelerometer used to measure moderate to vigorous physical activity (MVPA). The Actigraph calculates activity levels through predetermined cutpoints and has been validated against doubly labelled water techniques for estimating energy expenditure (*R = 0*.*3)* [7] and for step counts against the Yamax Digiwalker pedometer in free-living conditions with a 98.5% accuracy [9]. However, the Actigraph GT3X+ does not provide online user interaction or real-time outputs and is relatively cumbersome to wear at the hip, making it uncomfortable for participants to wear it at night [10]. The Actigraph also requires specific training for analysing data post-activity and an annual payment for a relatively expensive software license.

Globally, Fitbit has the largest market share for activity monitors, has sold more than 25 million devices and in 2015 had a revenue of US$1.8 billion [11]. The Fitbit Flex is a waterproof device that can be worn 24 hours a day as a wristband, which may be more convenient for users than the waist worn Actigraph. In addition, the Fitbit Flex provides access to an online database where users can view their activity outputs, join groups and interact with other users. Interaction with other users through social media platforms has been found to facilitate positive health behaviour changes through peer-support [12]. Moreover, real time feedback on physical activity levels can assist with self-monitoring. This has been demonstrated with the use of pedometers to be associated with a significant increase in physical activity, and a reduction in body mass index [13, 14]. Other health related data, such as sleep duration and nutritional breakdowns of user food logs, and estimation of kilocalories burned in response to activity, can also be viewed in combination with the physical activity information. The Fitbit Flex is a very popular consumer based activity tracker, is not very expensive, is user-friendly and has lots of other features that are interesting and motivating for users. However, before the Fitbit Flex can be used in a research setting as well, more information about the validity and reliability is needed. An earlier model of the Fitbit, the waist worn Fitbit One, was validated in laboratory conditions for treadmill walking against direct observation and showed substantial concordance (0.97–1.00) [15] and reasonable inter-device reliability (ICC = 0.90) [16]. However, few studies have assessed the validity and reliability of the wrist worn Fitbit Flex [17, 18].

Some studies have compared outputs from Fitbit activity monitors and the Actigraph, but with variability in protocols and device placement. Paul et al. [19] found excellent agreement in the average step count per day between the two devices recorded in free-living conditions and worn on the waist. Similarly, Gusmer et al. [20] found no significant differences in step counts between the Fitbit Ultra and the Actigraph G1TM, reporting that both devices can be used interchangeably to measure step count when they are both worn on the waist. Ferguson and colleagues [21] compared several consumer-level devices, including the waist worn Fitbit One, against the Actigraph GT3X+ and found strong validity for the Fitbit’s measurement of steps and MVPA. However, none of these studies compared free-living conditions and laboratory based activities across multiple outcome measures such as step count, MVPA and activity energy expenditure (AEE). Comparisons between several measures will give greater depth to existing knowledge in regards to validity of the device in potential field-based studies. The aim of the present study was to examine the validity and test-retest reliability of the Fitbit Flex against direct observation for measuring steps in the laboratory and against the Actigraph for step counts in free-living conditions and for MVPA and AEE overall.

## Methods

Twenty-five students (12 females, 13 males) were recruited through convenience sampling (word of mouth and noticeboard postings) from James Cook University in Cairns, Australia. All participants were free of illness and medical conditions that would contraindicate exercise. Participants provided written informed consent prior to participation in the study. The study was approved by the Human Research Ethics Committee of James Cook University (Approval number: H5763) and data were collected from June to August 2014.

Collection of demographic data and anthropometric measures was conducted onsite at the university in a private room. Baseline assessments included height, weight and body fat. Body fat was assessed using bioelectrical impedance analysis (Tanita body fat monitor/scale, BF-522, Tanita Corporation, Tokyo, Japan). Following baseline assessments, participants were fitted with an accelerometer (GT3X+, Actigraph, Florida, USA) on an elastic strap around the hips and a Fitbit Flex (Fitbit, Inc., San Francisco, USA) worn on the wrist. To maintain the same wear time, the participants were asked to remove both devices during water based activities.

The Actigraph GT3X+ accelerometer is a light weight device used to measure steps, moderate-to-vigorous physical activity and activity energy expenditure in free-living environments. The device was worn on participants’ preferred hip (just above the iliac crest) on an adjustable elastic belt during waking hours of a single day. During the initialisation of the device participant details were entered such as sex, height, weight, and age. The recording frequency was set to 30Hz and to measure activity in 60 second epochs. Moderate to vigorous physical activity was analysed using the Freedson equation using the cutpoints of 1952–5724 counts per minute (CPM) for moderate activity, 5725–9498 CPM for vigorous and >9498 CMP for very vigorous activity [22]. The data was downloaded using the ActiLife Data Analysis Software version 6.2.

The Fitbit Flex is also an accelerometer and records data using 60 second epochs. The device is worn on the wrist using a fitted band and can be worn 24 hours a day. Therefore, although the two devices are worn differently, they were both evaluated for reliability and validity in their optimal respective device placement. The wrist display of the Fitbit Flex uses five LED lights to represent quintiles of the daily goal which can be set as step count, distance or calories burnt. In our study, the daily goal was set to 10,000 steps but participants did not have access to online results. Complete data, including exact step count, minutes of MVPA and AEE, is available for users to access on the Fitbit website (www.fitbit.com).

Two data collection sessions in the laboratory were conducted for all study participants. There was missing data as a result of one participant failing to attend the second session, and one participant having a Fitbit device malfunction during the second session. This resulted in a total of 48 data samples for analysis. The treadmill based element of the exercise protocol required participants to be filmed undertaking six minute bouts of walking, incline walking, jogging and stair stepping. There was a four minute rest between the six minute bouts. Walking and jogging paces were self-selected using Borg’s Rate of Perceived Exhaustion scale (RPE) [23] for the first exercise session to estimate a walking pace with an RPE of 4 and an RPE of 7 for jogging. These speeds were exactly replicated for the second exercise session to assess test-retest reliability. The speeds for walking and jogging ranged from 5km/hr to 6.5km/hr and 8km/hr to 10km/hr respectively. The incline was set at 5% and the simulated stepping phase used the same 15cm high plyometric box for all participants, regardless of their height. Once the protocol was complete, participants were instructed to wear both devices for the remainder of the waking day. Since all the laboratory sessions were done in the morning hours, all participants had similar wear times for the measurements in free-living environments. However, the devices were not reset, nor was any data immediately recorded at the end of the session, so step counts, MVPA and AEE results from the free living conditions would include data from the laboratory session. A second laboratory session was completed within the same week in which the same protocol was repeated, and the walking and jogging speeds were matched to the self-selected speeds of the first session. The video footage from the laboratory sessions was reviewed by two persons who counted the number of steps. If the differences in the direct observations were greater than 10 steps, the footage was viewed a second time by both reviewers and this revised estimate was used. Data for direct observation was only available during the laboratory conditions, which included walking, incline walking, jogging and stair stepping. The step counts from both reviewers were averaged to create a single count for each activity. There were technical difficulties with video recording resulting in missing data for some activities for four participants in the first session and six participants in the second session. The availability of data for these participants by activity type and session for direct observation and the Fitbit and Actigraph devices is displayed in Table 1.

**Table 1:** Summary of direct observation, Actigraph and Fitbit at first and second measures and combined.

	Direct observation					Actigraph					Fitbit				
Activity	n	mean	SE	(95% CI)	C_v_	n	mean	SE	(95% CI)	C_v_	n	mean	SE	(95% CI)	Cv
**Walking step count**															
First measures	21	674.8	8.9	(656.3, 693.2)	6.0	25	726.1	9.0	(707.5, 744.7)	6.2	25	588.1	21.8	(543.1, 633.1)	18.5
Second measures	19	697.4	10.7	(674.9, 719.9)	6.7	23	722.6	9.7	(702.4, 742.8)	6.5	23	583.1	17.3	(547.3, 618.9)	14.2
Average of measures	23	684.0	10.0	(663.2, 704.8)	7.0	25	709.8	13.0	(683.0, 736.6)	9.1	25	583.6	16.1	(550.4, 616.8)	13.8
**Incline walking step count**															
First measures	22	691.0	9.2	(672.0, 710.1)	6.2	25	738.3	10.7	(716.3, 760.3)	7.2	25	643.3	22.2	(597.4, 689.2)	17.3
Second measures	21	701.1	10.6	(679.1, 723.1)	6.9	23	728.3	11.3	(704.9, 751.8)	7.5	23	652.9	17.4	(616.7, 689.0)	12.8
Average of measures	24	698.9	9.0	(680.3, 717.4)	6.3	25	714.2	19.3	(674.3, 754.0)	13.5	25	641.5	18.5	(603.2, 679.8)	14.5
**Jogging step count**															
First measures	23	945.9	9.8	(925.5, 966.3)	5.0	25	1009.4	13.2	(982.1, 1,036.6)	6.5	25	960.3	19.7	(919.5, 1,001.0)	10.3
Second measures	20	944.1	10.3	(922.6, 965.6)	4.9	23	1013.2	14.5	(983.1, 1,043.4)	6.9	23	977.6	18.3	(939.7, 1,015.5)	9.0
Average of measures	24	947.2	8.9	(928.8, 965.6)	4.6	25	1011.2	11.0	(988.5, 1,033.9)	5.4	25	971.1	14.7	(940.8, 1,001.5)	7.6
**Stair stepping**															
First measures	23	559.1	17.0	(523.9, 594.4)	14.6	25	578.6	24.1	(528.9, 628.2)	20.8	25	572.0	26.1	(518.1, 625.8)	22.8
Second measures	19	582.7	19.9	(541.0, 624.5)	14.9	23	538.8	23.3	(490.5, 587.2)	20.8	23	584.0	20.6	(541.3, 626.8)	16.9
Average of measures	23	569.2	16.9	(534.1, 604.3)	14.2	25	558.8	16.9	(523.9, 593.7)	15.1	25	575.3	17.9	(538.2, 612.3)	15.6
Energy expenditure–Calories*															
First measures	-	-	-	-	-	24	532.4	43.9	(441.7, 623.2)	40.4	24	818.2	69.0	(675.5, 960.9)	41.3
Second measures	-	-	-	-	-	23	563.1	58.1	(442.7, 683.6)	49.4	23	840.1	74.4	(685.7, 994.5)	42.5
Average of measures	-	-		-	-	25	538.9	38.8	(458.8, 619.0)	36.0	25	808.1	56.6	(691.3, 924.9)	35.0
Active minutes*															
First measures	-	-	-	-	-	24	53.8	4.0	(45.4, 62.1)	36.7	24	33.7	3.0	(27.4, 40.0)	44.3
Second measures	-	-	-	-	-	22	57.0	4.0	(48.8, 65.2)	32.6	22	38.1	3.2	(31.5, 44.7)	39.3
Average of measures		-	-	-	-	24	54.6	2.8	(48.7, 60.5)	25.5	24	35.4	2.4	(30.5, 40.3)	32.7

SE: standard error, CI: confidence interval, C_v_: Coefficient of variation

-: No data available

*: Comparisons are between the Fitbit and Actigraph

### Statistical Analysis

Step count data, AEE and MVPA are reported for the first and second laboratory data collections for the Actigraph and Fitbit devices (Table 1). The average of first and second step counts, AEE and MVPA were also derived for each participant. For the Actigraph and Fitbit devices, step count at first collection was used in place of the average for two participants who had missing second collection step counts. For direct observation, the first or second measure was used in place of the average depending on which of the two data collection points had missing data (n = 6).

Unlike the Actigraph, the Fitbit Flex does not just measure activity energy expenditure, but total energy expenditure. Hence, for comparability, activity energy expenditure results from the Fitbit were calculated by subtracting the basal metabolic rate (BMR) from the total daily energy expenditure. One participant’s data was excluded in the AEE analysis due to the total daily energy expenditure being less than the estimated basic metabolic rate. The distribution of step counts, AEE and MVPA in minutes were assessed using kernel density plots and Shapiro-Wilk tests for normality.

Coefficients of variation were calculated to describe the distribution of values for direct observation and both devices. Absolute differences and absolute proportional differences for step counts between each of the three measurement methods were calculated. Absolute proportional differences were calculated as the absolute differences between the Fitbit and direct observation, or the Fitbit and the Actigraph, divided by values for the Fitbit. Paired samples *t*-tests were also used to examine the differences in mean step count data between each of the three measurement methods at both data collection sessions and the derived average step counts of the two data collections. A *p* value <0.05 was considered significant for all analyses.

Intraclass correlation coefficients (ICC) were calculated to compare the reliability of the three measurement methods against each other during laboratory sessions and only for the Fitbit and the Actigraph during free living conditions. The type of ICC used was two-way mixed methods with absolute agreement. Bland-Altman plots comparing the three measurement methods were created for the average step counts and energy expenditure measures from the first and second data collections where applicable. The test-retest reliability between the first and second sessions for the three measurement methods was assessed using intraclass correlations, absolute differences and absolute proportional differences.

## Results

The mean age (± SD) of participants was 23.7±5.8 years. Females (n = 12) had an average height and weight of 165.6±5.8cm and 62.3±7.6kgs respectively and a BMI of 22.7±2.2 kg/m². Males (n = 13) were taller (175.7±4.4cm) and heavier (85.3±16.2kgs) and had a higher BMI (27.7±6.1 kg/m²). Body fat percentages for females and males were 26.3±4.8%, and 21.5±6.2% respectively.

Table 1 presents the distribution of step counts for direct observation and the Actigraph and Fitbit devices at first and second data collections and the average of these two collections. Energy expenditure and active minutes for each activity are also provided for the Actigraph and Fitbit. The greatest difference in step count was for walking, in which the average Fitbit estimate from both data collections ($\bar{x}$ = 583.6, 95%CI 550.4–616.8) was 15% lower compared to direct observation ($\bar{x}$ = 684.0, 95%CI 663.2–704.8). In contrast, the Actigraph overestimated step counts relative to direct observation for all activities except stair climbing, in which, similar to the Fitbit, it was also comparable to direct observation. Compared to direct observation and the Actigraph, the Fitbit generally had higher coefficients of variation for step counts. However, for stair climbing, the coefficient of variation was high for both devices. Outside of the laboratory, the Fitbit had lower estimates of free living average step counts (7,582.9±3,368.6 steps) compared to the Actigraph (10896.0±4,364.9 steps) and greater variation in step counts, as indicated by a slightly higher coefficient of variation (44.4 and 40.1 respectively). The Fitbit estimates for energy expenditure in calories were consistently higher compared to the Actigraph and the MVPA minutes were lower.

Table 2 displays the differences between the Fitbit and direct observation during laboratory stepping activities and between the Fitbit and Actigraph for free living outcomes, including steps, MVPA and AEE. In the laboratory, the largest differences between direct observation and the Fitbit were during the walking phase. When the results from sessions one and two were averaged, the absolute difference between these two measurement methods was over 100 steps ($\bar{x}$ = 110.3, 95%CI 74.3–146.3). As a proportion, this difference was 21% ($\bar{x}$ = 21.2, 95%CI 13.0–29.4) and a paired samples *t*-test for mean differences was significant ($\bar{x}$ = -104.0, 95%CI -143.5–64.5, *p*<0.001). There were similar differences for incline walking and stair climbing. For jogging however, the Fitbit was much more comparable to direct observation with a proportional difference of 6% ($\bar{x}$ = 6.4, 95%CI 3.7–9.0) across the two data collections.

**Table 2:** Comparisons and differences of direct observation and the Fitbit in the laboratory and the Fitbit and Actigraph in free living.

	Absolute differences		Absolute proportional differences		Paired samples *t*-test for differences			
Activity	Mean	(95% CI)	Mean	(95% CI)	Mean	SE	(95% CI)	*p*
**Walking step count**								
First measures	118.7	(77.3, 160.1)	24.5	(13.2, 35.8)	-93.4	25.7	(-147.1, -39.8)	0.0017
Second measures	129.5	(85.9, 173.1)	25.2	(15.2, 35.2)	-126.4	21.8	(-172.2, -80.7)	<0.001
Average of measures	110.3	(74.3, 146.3)	21.2	(13.0, 29.4)	-104.0	19.0	(-143.5, -64.5)	<0.001
**Incline walking step count**								
First measures	84.4	(42.9, 125.9)	16.8	(5.1, 28.6)	-56.9	24.1	(-107.1, -6.6)	0.0283
Second measures	75.6	(45.6, 105.6)	12.5	(7.1, 17.9)	-49.4	19.2	(-89.6, -9.3)	0.0183
Average of measures	77.0	(40.5, 113.4)	15.1	(4.7, 25.5)	-60.2	20.3	(-102.2, -18.3)	0.0068
**Jogging step count**								
First measures	66.4	(37.3, 95.5)	7.1	(3.6, 10.6)	21.1	19.4	(-19.1, 61.4)	0.2883
Second measures	83.2	(58.7, 107.6)	8.9	(5.9, 11.9)	22.9	21.7	(-22.7, 68.4)	0.3066
Average of measures	60.2	(38.2, 82.2)	6.4	(3.7, 9.0)	26.2	15.5	(-5.9, 58.3)	0.1052
**Stair stepping**								
First measures	104.9	(62.7, 147.0)	19.1	(11.6, 26.6)	15.8	30.0	(-46.5, 78.1)	0.6035
Second measures	78.7	(43.2, 114.3)	16.4	(5.9, 26.9)	-9.7	25.0	(-62.2, 42.9)	0.7031
Average of measures	86.6	(55.0, 118.2)	15.5	(10.1, 20.9)	3.0	23.9	(-46.6, 52.6)	0.9014
Free living steps*								
First measures	3855.3	(3,012.2, 4,698.3)	57.9	(46.1, 69.6)	-3851.8	409.8	(-4,697.7, -3,006.0)	<0.001
Second measures	3243.7	(2,074.4, 4,413.0)	44.6	(28.6, 60.5)	-3218.0	570.5	(-4,401.1, -2,034.9)	<0.001
Average of measures	3313.2	(2,462.0, 4,164.3)	47.2	(34.7, 59.6)	-3313.2	412.4	(-4,164.3, -2,462.0)	<0.001
Energy expenditure–Calories*								
First measures	294.8	(198.9, 390.7)	34.4	(27.3, 41.6)	285.8	48.8	(184.9, 386.6)	<0.001
Second measures	285.6	(198.9, 372.2)	32.0	(23.7, 40.3)	276.9	44.3	(185.0, 368.9)	<0.001
Average of measures	277.1	(195.1, 359.2)	32.4	(25.2, 39.6)	269.2	42.0	(182.6, 355.8)	<0.001
Active minutes*								
First measures	20.1	(14.6, 25.5)	82.2	(28.0, 136.4)	-20.08	2.6	(2,089.0, -25.5)	<0.001
Second measures	18.9	(13.5, 24.3)	57.0	(35.7, 78.3)	-18.91	2.6	(1,495.0, -24.3)	<0.001
Average of measures	19.1	(15.3, 23.0)	60.4	(43.2, 77.6)	-19.15	1.9	(5,029.0, -23.0)	<0.001

SE: standard error, CI: confidence interval

*: Comparisons are between the Fitbit and Actigraph

Outside of the laboratory there was high measurement discord between the Fitbit and the Actigraph (Table 2). For the average of both data collections, the absolute difference in free-living steps was over 3,000 or 47% as a proportion ($\bar{x}$ = 47.2, 95%CI 34.7–59.6). Similar proportional differences were seen for AEE and MVPA and all *t*-tests for mean differences were significant.

Table 3 displays the results of ICC analyses between direct observations and each of the two devices. The Fitbit had poor (*r* = 0.1–0.3) to moderate correlations (*r* = 0.3–0.5) with direct observation on average measures of step count activities, ranging from 0.01 for stair stepping (95%CI -1.48–0.59, *p* = 0.491) to 0.34 for jogging (95%CI -1.41–0.71, *p* = 0.145). In contrast, the Actigraph was significantly correlated with direct observation for almost all activities, with the only exception being stair stepping (0.42 95%CI -0.36–0.75, *p* = 0.104). Outside of the laboratory, the Fitbit and the Actigraph were highly correlated (*r* = 0.5–1.0) for total free-living steps (0.78, 95%CI -0.19–0.94, *p*<0.001) AEE (0.56, 95%CI -0.23–0.84, *p*<0.001) and MVPA (0.52, 95%CI -0.18–0.84, *p*<0.001) (data not tabled).

**Table 3:** Intraclass correlations between Fitbit and Actigraph and between devices and direct observations.

	ICC Actigraph vs direct observation			ICC Fitbit vs direct observation			ICC Fitbit vs Actigraph		
	Average ICC	(95% CI)	*P*	Average ICC	(95% CI)	*p*	Average ICC	(95% CI)	*p*
**Walking step count**									
First measures	0.67	(-0.23, 0.90)	<0.001	0.11	(-0.48, 0.55)	0.353	0.09	(-0.23, 0.43)	0.298
Second measures	0.75	(0.29, 0.91)	0.001	0.00	(-0.29, 0.37)	0.503	0.16	(-0.17, 0.50)	0.097
Average of measures	0.73	(-0.14, 0.91)	<0.001	0.05	(-0.30, 0.42)	0.390	0.10	(-0.15, 0.39)	0.195
**Incline walking step count**									
First measures	0.75	(-0.17, 0.93)	<0.001	0.07	(-0.82, 0.57)	0.421	-0.06	(-0.66, 0.42)	0.583
Second measures	0.71	(0.31, 0.88)	0.002	0.19	(-0.58, 0.63)	0.281	0.21	(-0.33, 0.60)	0.202
Average of measures	0.74	(0.07, 0.91)	<0.001	0.11	(-0.58, 0.56)	0.356	0.02	(-0.45, 0.43)	0.467
**Jogging step count**									
First measures	0.57	(-0.20, 0.84)	0.001	0.40	(-0.39, 0.74)	0.116	0.58	(0.08, 0.81)	0.008
Second measures	0.35	(-0.25, 0.71)	0.035	0.11	(-1.22, 0.65)	0.399	0.38	(-0.34, 0.73)	0.116
Average of measures	0.46	(-0.23, 0.78)	0.005	0.34	(-0.41, 0.71)	0.145	0.52	(-0.01, 0.78)	0.018
**Stair stepping**									
First measures	0.18	(-1.01, 0.66)	0.329	-0.06	(-1.63, 0.56)	0.553	0.69	(0.28, 0.86)	0.004
Second measures	0.53	(-0.10, 0.81)	0.041	0.51	(-0.32, 0.81)	0.077	0.43	(-0.26, 0.75)	0.085
Average of measures	0.42	(-0.36, 0.75)	0.104	0.01	(-1.48, 0.59)	0.491	0.72	(0.38, 0.88)	0.001

CI: confidence interval, ICC: Intraclass correlation

**Fig 1:**
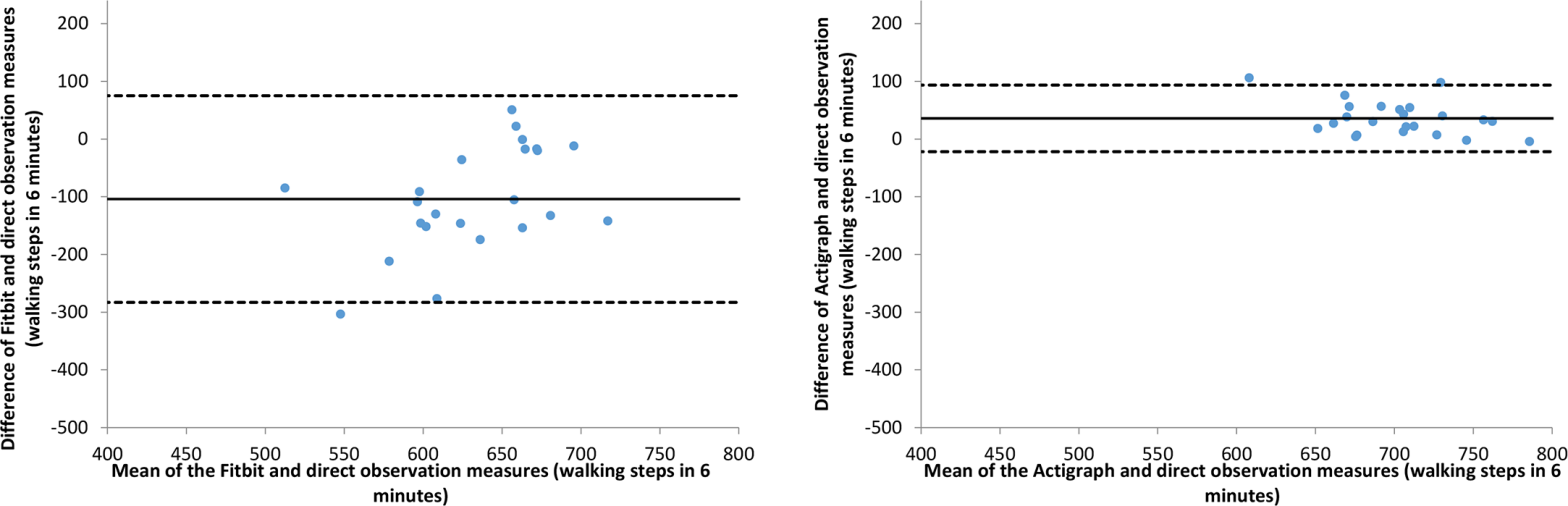
Bland-Altman plots of walking steps for the Fitbit and Actigraph compared to direct observation.

**Fig 2:**
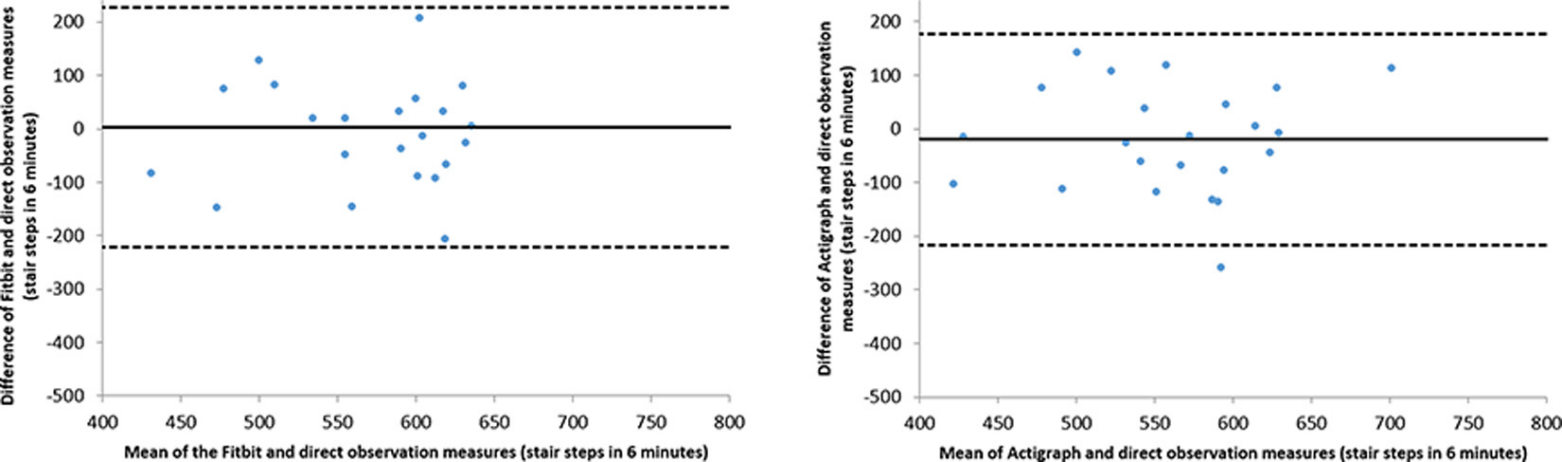
Bland-Altman plots of stair steps for the Fitbit and Actigraph compared to direct observation.

Bland-Altman plots demonstrated the Fitbit had a high level of measurement discord with direct observation relative to the Actigraph. For almost all of the laboratory activities, as the mean steps measured by the Fitbit and direct observation increased, the differences between these measurements also increased. During walking, the Fitbit underestimated steps for small amounts of activity and became increasingly accurate as the mean number of steps increased (Fig 1). Within a six minute period, the Fitbit may estimate as much as 283 steps lower and up to 75 steps higher than direct observation. In contrast, the Actigraph had much less measurement discord, with the lower and upper limits for step estimates as -22 and 94 respectively. This contrasting accuracy between the Fitbit and Actigraph held for all laboratory activities, with the exception of stair climbing. For this activity, both devices had high and comparable levels of measurement discord with direct observation (Fig 2).

**Fig 3:**
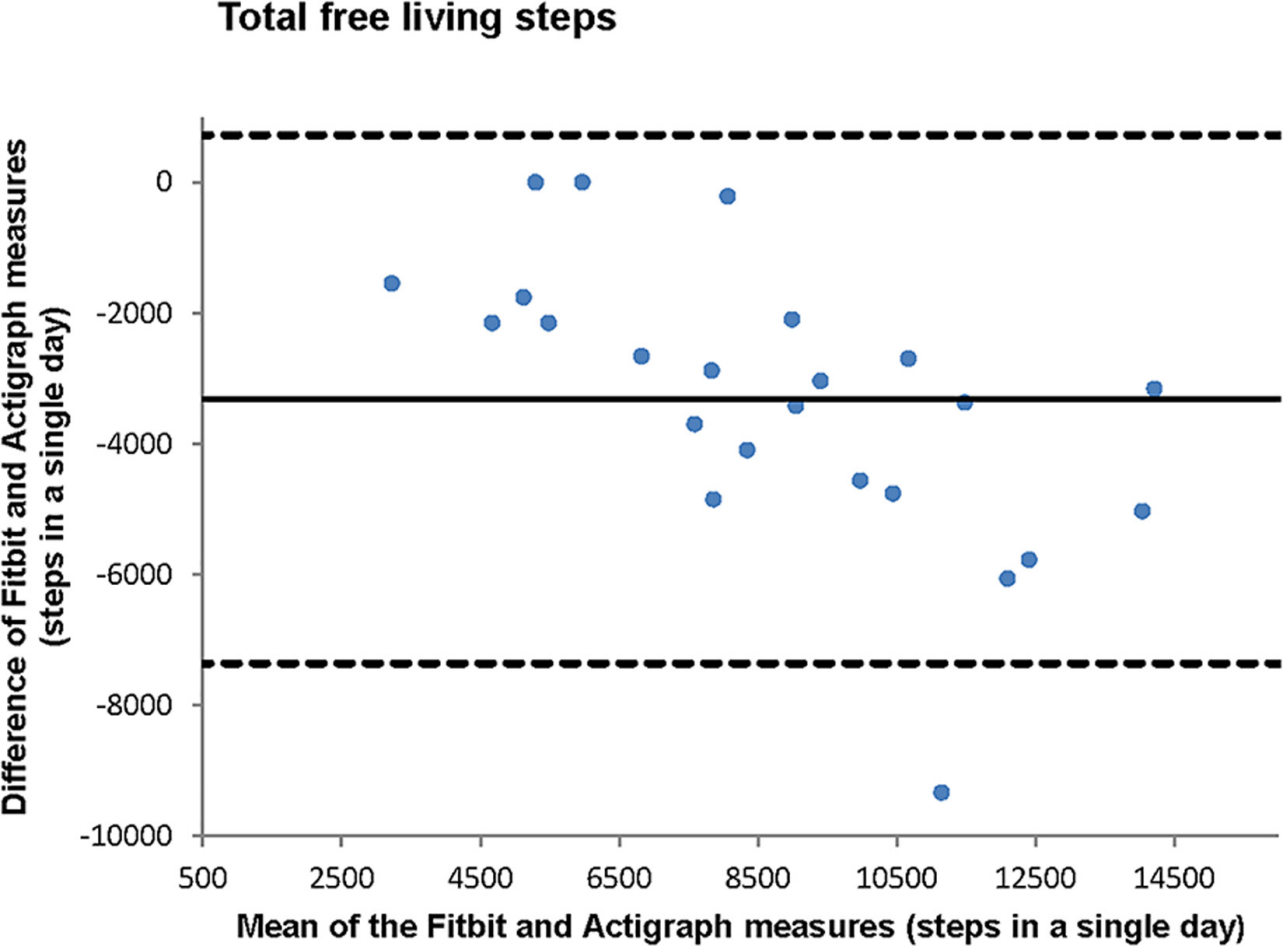
Bland-Altman plot of free living steps for the Fitbit compared to the Actigraph.

Outside of the laboratory, Bland-Altman analyses indicated there was high measurement discord between the Fitbit and Actigraph for free-living steps (Fig 3). The Fitbit tended to undercount steps compared to the Actigraph and this underestimate grew stronger as the total number of steps increased.

In terms of test-retest reliability, the mean absolute difference in steps for the Fitbit between session 1 and 2 ranged from 71.9 for walking to 83.1 for incline walking. As mean proportions, these differences were 13.6% (95%CI 4.6–22.6) and 12.3% (95%CI 7.8–16.9) respectively. In contrast, the mean absolute proportional differences for the Actigraph on the same measures were lower, at 4.8% (95%CI 2.8–6.8) and 4.7% (95%CI 2.7–6.7) respectively. Stair climbing was the exception to this trend, with both the Fitbit and Actigraph having mean proportional differences of around 20% between first and second data collections. For direct observation, the mean proportional difference for stair climbing was 10.6% (95%CI 4.4–16.7) with a mean absolute difference of 56.8 steps.

Test-retest reliability analyses using intra-class correlations indicated the Fitbit had greater variation between the first and second data collections relative to the Actigraph for most of the laboratory activities (data not tabled). Between the two laboratory sessions, the Fitbit had a moderate correlation for the walking activity (ICC = 0.57, 95%CI: -0.02,0.82, *p* = 0.028), as did the Actigraph (ICC = 0.59, 95%CI: 0.03,0.83, *p* = 0.022). The Fitbit had modest and non-significant correlations for incline walking (ICC = 0.22, 95%CI: -0.92,0.68, *p* = 0.288 and jogging (ICC = 0.26 95%CI:-0.73,0.69, *p* = 0.239). On these measures, the Actigraph performed somewhat better between the two sessions, with a strong correlation for incline walking (ICC = 0.75, 95%CI: 0.41,0.89, *p* = 0.001) and a moderate agreement for jogging (ICC = 0.49, 95%CI: 95%CI: -0.23,0.78, *p* = 0.067). Both the Fitbit and Actigraph had low and non-significant agreement for stair stepping between first and second laboratory sessions with ICCs of 0.34 and 0.19 respectively.

## Discussion

### Summary of Principle Findings

We compared results from the Fitbit Flex against direct observations of step counts in a laboratory setting and free-living and overall steps, moderate to vigorous activity and activity energy expenditure against the Actigraph. The results from our study indicated that the Fitbit has moderate validity relative to direct observation and the Actigraph. It tended to vary between over-counting and under-counting steps, depending on the activity type. Our findings contrast a previous study which utilised the same device placement and compared the step count outputs of the Actigraph against the Fitbit Flex over a seven day period [24]. In that study there were no significant (*p* = 0.052) differences between the devices, however, the differences in the findings could be due to variation in study protocols. Dierker and Smith [24] compared step counts between five activity monitors, using a protocol that advised participants to remove the devices during exercise. This essentially would not capture all free-living activity and encompassing different exercise modalities that were assessed in our study. In the present study, the mean MVPA minutes were lower in the Fitbit (35.4) compared to the Actigraph (54.6), but AEE was greater from the Fitbit (808.1 calories) compared to the Actigraph (538.9 calories). In terms of test-retest reliability across the two sessions, the Fitbit had greater variation in step count estimates compared to the Actigraph for most of the laboratory activities.

### Implications of Findings

The Bland-Altman plots showed that the magnitude and direction of step count difference between these devices varied depending on the type and volume of activity. This indicates that the divergence in terms of the Fitbit Flex step count between direct observations is not constant and is affected by the type of activity and the number of steps taken, which is also evident in the weak to moderate intraclass correlation coefficients and test-retest reliability. Previous work has indicated that the hip worn Fitbit Ultra is reliable and valid for activity monitoring in terms of flat surface step count, but is not recommended for incline activity [25]. In our study, there were significant differences in step counts between direct observation and the wrist worn Fitbit Flex during treadmill walking (*p*<0.001) and incline walking (*p* = 0.007) which suggests that the Fitbit Flex may have low validity for low to moderate intensity walking and walking on an incline. Due to variations in fitness levels, the participants self-selected the treadmill speeds, which was not optimal as the device validity was dependant on the treadmill speed. Two non-significant differences were obtained during jogging and the stair stepping activity. The higher accuracy in step counts of stair stepping, may have been due to participants stepping up and down a box, having a more pronounced arm movement.

Free living step counts in our study were significantly underestimated by the Fitbit Flex compared to the Actigraph. The more steps, the greater the underestimation which is seen in the limits of agreement, this could be due to the variability in movements by participants in free-living conditions and continuous undercounting in low intensity activities. In our study the Fitbit recorded consistently higher AEE with a 50% higher average measure (808.1±282.9 calories) compared to the Actigraph (538.9±194.0 calories). This is reflected in the correlation tests between the devices, with the average of the two protocol measures for AEE only having a moderate agreement (ICC = 0.56). The findings in regards to AEE need to be interpreted with caution, due to the differences in measurement equations. As mentioned before, the Actigraph only records AEE, whereas the Fitbit records total energy expenditure based on the sum of the AEE and the BMR. The BMR is calculated using the Mifflin-St Jeor equation, which requires participants’ gender, height, and weight.

In contrast to the higher AEE, the average of the MVPA minutes in the Fitbit (35.4 ± 11.8) was significantly lower compared to the Actigraph (54.6 ± 13.9) and the coefficient of variation was greater in the former device (32.7% and 25.5% respectively). Some of the variation in outcome measures may be due to different criteria cutpoints for moderate to vigorous physical activity minutes. Moreover, the Fitbit only records MVPA minutes if the intensity is over 3 METs and, in line with physical activity guidelines [26, 27], only in bouts of at least 10 minutes. In contrast, the Actigraph uses predetermined counts per minute cutpoints to determine activity intensity and duration, including bouts of as little as one-second epochs.

The test-retest reliability analyses in this study indicate the step count estimates produced by the Fitbit over multiple time points would vary more in terms of absolute proportional differences compared to the Actigraph. When compared to direct observation, the Fitbit can overestimate step counts for jogging, but also underestimate the number of steps for walking. While both devices had moderate reliability for estimating flat walking steps in the laboratory environment based on ICCs, only the Actigraph produced reliable incline walking step estimates. Neither device provided reliable stair walking steps estimates, however, even by direct observation there was variation in step count between sessions on this activity, which indicates that participant performance may have been responsible for at least some of the variation. The intraclass correlations were not reported for the free-living outcome measures as there may have been variations in the participants’ routine over the duration of the two days of assessment.

The placement of the devices is important as recent research indicates that wrist worn accelerometers might not be as accurate as waist worn devices in counting steps [28, 29]. For example, in activities such as washing dishes, the body can be in a fixed position, but arm movements are occurring. On the other hand, there is the potential for error as activities may also require steps to be taken, but the arms having minimal movement, for example carrying a box [30]. A recent study reported that the wrist worn Fitbit Flex and Nike Fuelband undercounted steps compared to devices worn on the waist or worn in the pockets of the participants’ pants [29]. However, wrist worn devices can avoid decreases in wear time compliance as the burden of having to continually remove equipment for water-based activities and to sleep would not be an issue [31].

The Fitbit’s accuracy in the measurement of jogging and stair stepping has been confirmed by this research, but more work is needed to validate lower intensity activities, such as walking. Consumer level devices allow for monitoring of health behaviours by providing immediate feedback via displays and internet-based applications. Participant reactivity needs further examination when using fitness monitors that provide immediate feedback to users. A study from van Hoye et al. [14] found reactivity with the use of pedometers caused a significant increase compared to baseline assessments in step counts during the first week of an intervention. There were no significant increases in step count after the first week of the intervention, which suggests that there is a ceiling effect in regards to device reactivity and step count [14]. A systematic review of 26 studies found that with the use of feedback from a pedometer users increased their activity levels by 27% compared to baseline levels [13]. This was primarily achieved by using the pedometer for rapid feedback to inform goal setting and realistic daily physical activity targets. However, reactivity to feedback is a consideration for short term interventions that allow users to access their data online [14]. As mentioned previously, in the present study the participants did not have access to detailed online data on their activity from the Fitbit website. The only feedback they got were the five LED lights on the Fitbit Flex that represent quintiles of the target step count, which would have limited reactivity. However, the provision of multiple measures, such as step counts, activity intensity and duration, might assist laypersons in becoming aware of their own physical activity levels, and allow comparisons against the current physical activity guidelines [32].

### Limitations

In order to ease participant burden, the wear time was limited to the study protocol and several hours of free-living. As mentioned previously, in order to have the same wear times, the participants were asked to remove both devices during water based activity. This could be considered as a theoretical limitation as participants might have forgotten to put the devices back on immediately after water-based activities, although unlikely in this study due to the short wear time. The data on AEE and MVPA from the laboratory session were carried over into the free living conditions, which may have impacted the results, as it was not a true representation of only free-living activity. Device compatibility was another issue that potentially limited this study as both devices utilise different equations and cutpoints for the data outputs.

## Conclusions

Our findings suggest that the Fitbit Flex has moderate validity for measuring physical activity relative to direct observation and the Actigraph. The test-retest reliability between the two sessions indicated greater variation with the Fitbit relative to the Actigraph. Poor test-retest reliability could have been a result of the inaccuracy of the Fitbit with low intensity activities. The Fitbit might be more suitable in studies in which immediate feedback through detailed data online is needed and where cost is an issue, but not necessarily in clinical settings. For selecting the appropriate physical activity monitor for research projects investigators should consider the activity domains involved, sample sizes, as well as the budget and participant burden [33]. Physical activity surveillance studies using the Fitbit Flex should consider the potential effect of measurement reactivity and undercounting of steps.

## Supporting Information

S1 File Dataset. Sections: 1 Device output step count, 2 Direct observation, 3 Activity minutes & energy output.
(XLSX)
